# Controlling the emission properties of solution-processed organic distributed feedback lasers through resonator design

**DOI:** 10.1038/s41598-019-47589-4

**Published:** 2019-08-01

**Authors:** Víctor Bonal, José A. Quintana, José M. Villalvilla, Pedro G. Boj, María A. Díaz-García

**Affiliations:** 1 Dpto. Física Aplicada, Instituto Universitario de Materiales de Alicante y Unidad Asociada UA-CSIC, Universidad de Alicante, 03080 Alicante, Spain; 2 Dpto. Óptica, Farmacología y Anatomía, Instituto Universitario de Materiales de Alicante y Unidad Asociada UA-CSIC, Universidad de Alicante, 03080 Alicante, Spain

**Keywords:** Lasers, LEDs and light sources, Electronic devices, Lasers, LEDs and light sources

## Abstract

Surface-emitting distributed feedback (DFB) lasers with both, resonator and active material based on solution-processable polymers, are attractive light sources for a variety of low-cost applications. Besides, the lasers should have competitive characteristics compared to devices based on high-quality inorganic resonators. Here, we report high performing all-solution-processed organic DFB lasers, consisting of water-processed photoresist layers with surface relief gratings located over the active films, whose emission properties can be finely tuned through resonator design. Their laser threshold and efficiency are simultaneously optimized by proper selection of residual resist thickness and grating depth, *d*. Lowest thresholds and largest efficiencies are obtained when there is no residual layer, while a trade-off between threshold and efficiency is found in relation to *d*, because both parameters decrease with decreasing *d*. This behaviour is successfully explained in terms of an overlap factor *r*, defined to quantify the interaction strength between the grating and the light emitted by the active film and traveling along it, via the evanescent field. It is found that optimal grating depths are in the range 100–130 nm (*r* ~ 0.5−0.4). Overall, this study provides comprehensive design rules towards an accurate control of the emission properties of the reported lasers.

## Introduction

Organic distributed-feedback (DFB) lasers, consisting of an organic active film and a relief grating as laser resonator, have received great attention in the last years^1,2^ for their potential applications in different areas, such as spectroscopy^3^, optical communications^4^ and sensing^5–7^. DFB lasers present various advantages with respect to other type of lasers: they can provide narrow single mode emission (linewidth < 1 nm), they show a low threshold (i.e. minimum pump intensity to operate), they can be prepared by low-cost methods and easily integrated with other devices.

Many advances have been achieved in the field of organic DFB lasers in relation to different aspects, such as decreasing the laser threshold, improving the photostability, obtaining a wide wavelength tunability range, etc^1^. However, most state-of-the-art devices have been based on high-quality DFB gratings engraved on conventional inorganic substrates (e.g. glass or SiO_2_) often prepared by sophisticated methods. So, nowadays many efforts are focusing on obtaining high performing lasers, in which both, the resonator and the active film, are polymeric^8–16^. This way, devices can be mechanically flexible and can be prepared in an inexpensive manner by solution-based methods. Another important aspect is to obtain wavelength tunability in a single chip. Although various strategies to achieve this aim have been proposed, they are often based on sophisticated technologies and generally it is not possible to keep a good threshold performance for the whole tunability range^17–19^.

**Figure 1:**
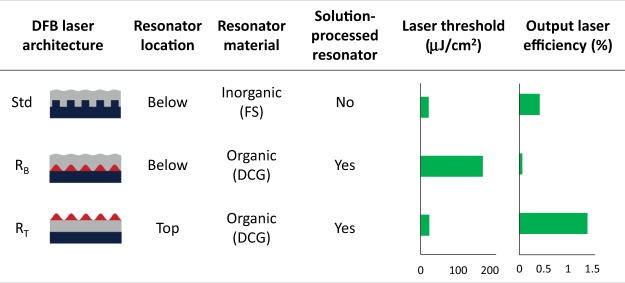
Relevant properties of different DFB laser architectures. Std, Standard, with resonator engraved on inorganic fused silica (FS) substrate below the active film; R_B_, dichromated gelatin (DCG) resonator below the active film; R_T_, DCG resonator on top of the active film. Data obtained from ref.^13^.

In this context we recently reported solution-processed DFB lasers with top-layer polymeric resonators showing an excellent laser performance in comparison to lasers with other types of DFB configurations (see Fig. 1)^13^. The reported top-layer resonator devices (configuration denoted as R_T_), showed low thresholds (~10 μJ/cm^2^, or 1 kW cm^−2^) and long operational lifetimes (7 × 10^5^ pump pulses), comparable to those of lasers based on the same active materials but with the gratings engraved on inorganic substrates (configuration denoted as standard, Std), and much better (by around one order of magnitude) than those with the polymeric resonator located below the active film (denoted as R_B_). The better performance of the R_T_-type devices, relative to that of standard ones, is in agreement with simulations based on the finite element method^8^. According to them, the former type is less sensitive to defects or additional modulation of the grating and besides, the active film volume is more efficiently used, which results in better oscillation modes. With regards to R_B_-type devices, their larger thresholds are a consequence of the low refractive index contrast between the active film and the resonator layer^12,13,15^. Another remarkable advantage of R_T_-type devices is their much superior laser efficiency. This is because the laser resonator, besides providing feedback for the light traveling along the waveguide, also constitutes the mechanism to extract the light out of the device and this is much more efficient when the grating is located on top. They key for the successful performance of the R_T_-type device is its architecture: a resonator, consisting of a water-soluble dichromated gelatin (DCG) photoresist layer with a holographically engraved one-dimensional (1D) relief grating, deposited on top of an active film of a thermoplastic polymer doped with an efficient laser dye. An important feature is that the thickness of the active film is constant across the device because the diffractive grating that provides the feedback required for laser emission is in a separated layer. This, together with the fact that the holographic lithography (HL)^20^ technique enables the preparation of large samples (of various centimetres), allowed fabricating a multi-colour emission device that kept a low threshold value at any wavelength^13^. Moreover, the fact that the DCG layer is processed from water is important because the properties of the active layer keep unaltered by the resonator preparation on top of it.

So far, the proposed photo-lithographic method has been only used to fabricate DFB resonators for organic lasers with different architectures^10–16^. But it shows promise for other optical nanoscale cavity geometries, for lasers or for other types of optoelectronic devices, and with other types of active materials. For example, with perovskite materials, which have shown excellent optical properties for a wide variety of devices such as solar cells^21^, light-emitting diodes^22,23^, lasers^24–26^, and nonlinear optical devices^27^. At this respect, the preparation of nanostructured hybrid perovskite media is a very interesting approach towards novel designs for devices with improved performance^28,29^.

In the present work we report a detailed investigation of the influence of the grating depth and the thickness of the residual layer on the laser performance of top-layer DCG resonator DFB lasers. In addition, the relationship between these geometrical parameters and the most important parameters of the holographic fabrication process (the initial thickness of the photoresist layer, i.e. before the grating recording; and the development time) is also presented. It is known, from studies on DFB lasers with other types of architectures^30–32^, and more generally on other types of optoelectronic devices, such as light-emitting diodes^33^, that such investigations are essential to achieve a fine control for the design and fabrication of devices to present the desired properties and ultimately to optimize their performance. Note that in our previous study of lasers with top-layer resonators, which aimed at revealing the characteristics of the top-layer resonator geometry in comparison to others, all the experiments were performed with resonators of similar geometrical parameters^13^. Another novel aspect of the present study, related to device processing, is that the dry development process performed on the DCG layer, after exposure, to generate the relief grating, is done here in a very simple and cheap way, i.e. by means of an inexpensive plasma cleaner machine, working at a moderate vacuum pressure. This is in contrast with the much more complex system used previously: an oxygen electron cyclotron resonance stream inside a high vacuum chamber^13^.

## Results and Discussion

### Fabrication of DFB lasers: relationship between fabrication and geometrical resonator parameters

**Figure 2:**
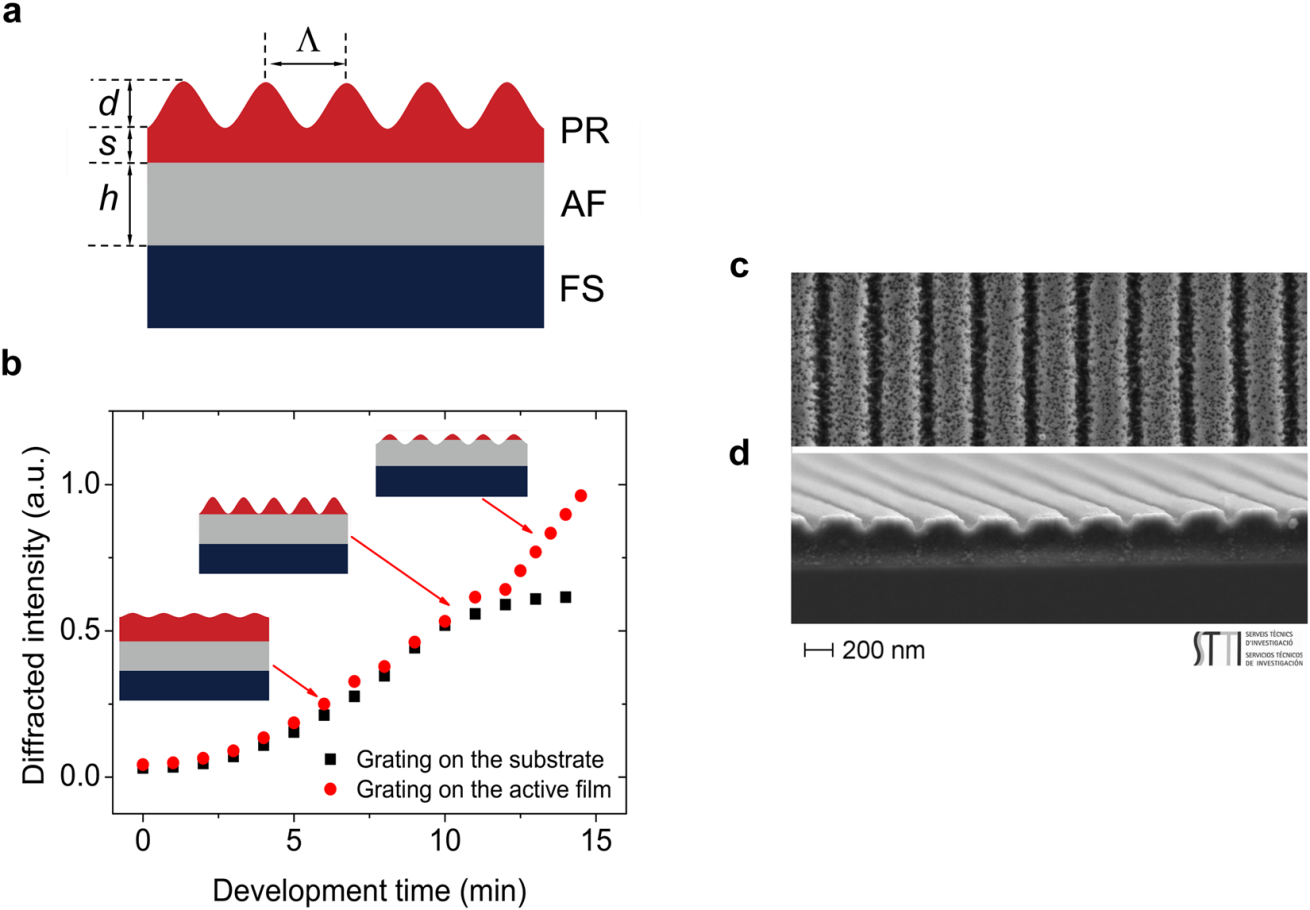
DFB laser architecture and influence of fabrication parameters. (**a**) Scheme of a top-layer resonator organic DFB laser, consisting of a photoresist (PR) layer with a surface relief grating (*d*: modulation depth; Λ: period; *s*: residual layer) deposited over an active film (AF) (*h*: thickness) prepared over a fused silica (FS) substrate. (**b**) Development curves (diffracted intensity versus the development time, *t*_D_) corresponding to a DCG PR layer coated over an AF (circles) and directly over FS (squares). Inset drawings illustrate different cases: grating with residual layer (at *t*_D_ ~ 6 min), grating with no residual layer (at *t*_D_ ~ 10.5 min) and grating imprinted on the active film (at *t*_D_ ~ 13 min); (**c**,**d**) Top and lateral FESEM images, respectively, of a DCG grating with period Λ = 372 nm, depth *d* ∼ 120 nm, and residual layer thickness *s* ∼ 260 nm.

The device structure (Scheme in Fig. 2a), includes as resonator a DCG photoresist layer with a 1D relief grating (*s*: thickness of the residual photoresist layer; *d*: grating modulation depth), deposited on top of an active film (*h*: thickness) of PS doped with perylene orange as laser dye.

All the DFB devices prepared in this work operate in the second order of diffraction, that is *m* = 2 in the Bragg condition (Equation 1)^1,2^

1 $$m{\lambda }_{{\rm{Bragg}}}=2{n}_{{\rm{eff}}}{\rm{\Lambda }}$$

where *n*_eff_ is the effective refractive index of the waveguide (which depends on *h*, as well as on the refractive indexes of the film, substrate and cover) and Λ is the grating period. In second-order DFBs light is coupled out of the film mainly in a direction perpendicular to the film by first-order diffraction, at a wavelength *λ*_DFB_ close to *λ*_Bragg_. Considering light traveling in a given waveguide mode, coupled mode theory^34^ predicts that for pure index gratings (this is the case for the lasers prepared here, because *h* is uniform across the device and the grating is in a separated layer), the wavelength that exactly satisfies Eq. (1) cannot propagate in the film. So, a photonic stop-band centered at *λ*_Bragg_ appears, and lasing oscillates on a pair of wavelengths, one at either edge of the dip. However, in the case of second-order devices, the peak with the lower wavelength has a larger threshold due to radiation losses^35^. Accordingly, single-mode emission at the peak of the longer wavelength is observed.

The two geometrical resonator parameters analysed in this work (Fig. 2a), thickness of the residual layer (*s*) and grating modulation depth (*d*) depend on two fabrication parameters: the initial thickness of the DCG layer (*s*_0_) and the development time (*t*_D_).

For a given *s*_0_ value, as *t*_D_ increases, *d* grows and consequently the diffraction efficiency (*η*) of the grating. This is illustrated in Fig. 2b which shows the evolution of the diffracted intensity measured during the development process, as a function of *t*_D_ for *s*_0_ = 100 nm. Results for a film deposited directly over fused silica (instead of over an active film, as general for all the DFB devices) are also included in Fig. 2b. Their purpose is to provide a way to determine the development time at which the residual layer is eliminated (*s* = 0). This corresponds to that at which *η* no longer increases and reaches a plateau. Note that for the film deposited over the active film, *η* keeps increasing even after the residual layer has been eliminated. This is because the development process also affects the active film, so *d* keeps increasing because a grating is formed in the active film itself. Such situation would correspond to devices with a different architecture (i.e. with imprinted active films)^36–38^, in which the active film thickness would no longer be uniform across the device.

Figure 2c,d show top and lateral field effect scanning electron microscope (FESEM) images, respectively, of one of the fabricated gratings (with *d* = 120 nm and *s* ∼ 260 nm). Remarkably, grating quality and morphology are similar to those of gratings fabricated in previous studies with a much more sophisticated development process than the one used in the present work^13^. The grainy texture of the surface is produced by gelatin fibres randomly distributed but oriented rather parallel to the substrate surface^39^. Such texture is observed independently on the type of development process. After developing, the grating profile is not sinusoidal, indicating that the development process is a nonlinear function of exposure. With this procedure the duty cycle for all the gratings fabricated is approximately 75:25 (hill:valley) so that a high feedback coupling coefficient, i.e. a low threshold, can be achieved^40,41^.

### DFB laser performance: dependence on the resonator geometrical parameters

In order to analyse the effect on the laser performance of *s* and *d*, we prepared four sets of DFB devices with *s*_0_ values of 50, 100, 240 and 400 nm. By considering that the development selectivity is approximately 10, the maximum achievable *d* for each set, corresponding to *s* = 0 and with no grating engraved on the active film, would be around 45, 90, 216 and 360 nm, respectively.

**Figure 3:**
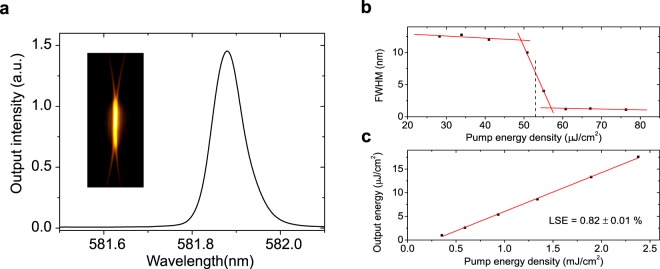
Example of laser characterization of a DFB laser. Properties of a device with grating modulation *d* = 260 nm and residual layer *s* = 110 nm. (**a**) Spectrum; the inset shows the corresponding image of the emitted light. (**b**) Linewidth, defined as full width at half maximum (FWHM), vs. the pump pulse energy, for laser threshold determination. (**c**) Output energy vs. pump energy, for laser efficiency (*LSE*) determination.

A complete laser characterization was performed for all the prepared devices, obtaining their emission wavelength (*λ*_DFB_), linewidth (FWHM), threshold (*E*_th_) and laser slope efficiency (*LSE*). Results for one of them are shown in Fig. 3. Single mode emission with a linewidth < 0.1 nm was obtained in all cases. For all the DFB lasers prepared, it is seen that the emitted laser light is linearly polarized, in a direction parallel to the grating lines. This indicates that the laser mode is associated to the fundamental transverse electric waveguide mode TE_0_. Also, the beam divergence observed in a direction perpendicular to the grating lines is ~5 · 10^−3^ rad.

### Effect of varying *s* and *d* on the *E*_th_ and *LSE* laser parameters

**Figure 4:**
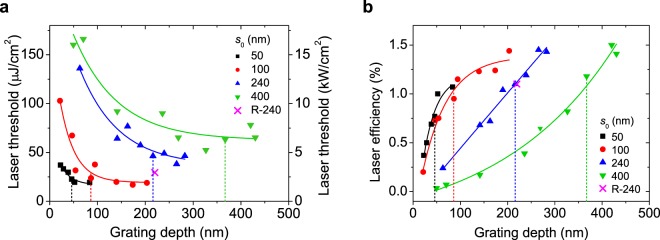
Influence of the grating depth on the laser threshold and efficiency. (**a**) Laser threshold, expressed as pump energy density and power density (left and right axis, respectively; error ~ 10%); and (**b**) laser efficiency (error ~ 10%) vs grating depth for four series of DFB lasers with different values of the initial resist layer thickness, *s*_0_ (see legend). The full lines are guides to the eye. Dashed lines indicate, for each series, the device with no residual layer (*s* = 0). For all the lasers, a simple grating development system was used. Data for a laser (denoted as R-240, see legend) with *s*_0_ = 240 nm and prepared by using a much more complex anisotropic dry development system are shown.

The influence of changing *d* and *s* on the threshold and *LSE* of all the prepared devices is analysed through Fig. 4a,b, respectively, in which data are plotted as a function of *d*. For a given set (each with a different *s*_0_ value), *s* decreases as *d* increases, according to *s*∼*s*_0_ − (10/9) *d*. Data for the devices with no residual layer (*s* = 0) can be easily located by the vertical dashed lines shown.

With regards to the threshold (Fig. 4a), a similar behaviour is observed for the four series of experiments: the threshold decreases with increasing *d* (and therefore decreasing *s*), first at a high rate and then at a slower rate, reaching a minimum value when *s* = 0. By comparing the minimum thresholds for each series, the lowest values (~23 μJ/cm^2^) correspond to those with smaller *d* (46 and 86 nm), whose *s*_0_ values are 50 and 100 nm, respectively. Larger thresholds (46 and 63 μJ/cm^2^) are obtained for devices with larger *d* and *s*_0_ values (*d* = 216 nm and *s*_0_ = 240 nm; and *d* = 367 nm and *s*_0_ = 400 nm, respectively). Note that for some devices, i.e. those with *s* < 0, the development times were sufficiently long as to have the gratings imprinted directly on the active film. For such lasers, the thresholds are similar than those obtained when *s* = 0.

With respect to the effect of varying *s* and *d* on the *LSE* (Fig. 4b), it is seen that *LSE* increases with increasing *d*. Although this increase continues after the residual layer has been eliminated (after that, the grating starts getting imprinted on the active film), its rate diminishes for the two series of lasers with lower thresholds (those with lower *s*_0_, 50 and 100 nm, and therefore lower *d*, for *s* = 0; Fig. 4a). Note that among these two series, somewhat better efficiencies are obtained for the one with *s*_0_ = 100 nm.

All the lasers prepared had gratings developed with a simple process, as explained in the methods section. In order to assess the importance of using this type of development process, in comparison to the more sophisticated one used in previous studies (an oxygen electron cyclotron resonance stream inside a high vacuum chamber), data for a laser with *d* = 220 nm and *s* = 0, prepared with this latter method, have been included in Fig. 4a,b (denoted as R-240). Considering the accuracy of measurements (~10%), no differences are observed in the *LSE*, while the threshold intensity is somewhat lower in the device prepared with the anisotropic method.

**Figure 5:**
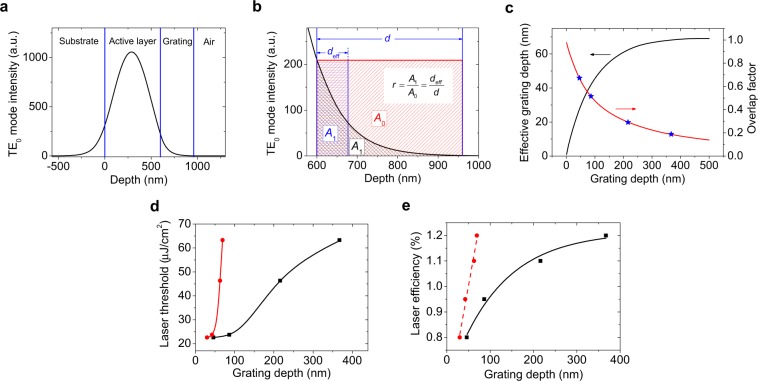
Analysis of the laser threshold and efficiency results in terms of the interaction of the evanescent wave of the light traveling along the active layer with the grating. (**a**) Electric field intensity distribution of the fundamental transverse electric (TE_0_) mode for a laser with *s* = 0, *s*_0_ = 400 nm and *d* = 367 nm. (**b**) Electric field intensity distribution corresponding to the grating to illustrate the definition of the overlap factor *r* through the equation (1) shown as an inset (*A*_1_: area under the curve in the grating corrugation region; *A*_0_: area of rectangle with a width equal to *d* and height equal to electric field intensity at the active layer surface; *d*: grating depth; *d*_eff_: effective grating depth). (**c**) Effective grating depth (black line, left axis) and overlap factor (red line, right axis) as a function of grating depth. Overlap factors for lasers without residual layer (*s* = 0) are indicated with blue stars. (**d**) Laser threshold energy density and (**e**) laser efficiency as a function of grating depth (black full squares) and of effective grating depth (red full circles) for lasers without residual layer (*s* = 0). Dashed line in (**e**) is a linear fit to data. Other lines relating points in (**d**,**e**) are guides to the eye.

The threshold and *LSE* results are here analysed by considering the interaction between the grating and the evanescent wave of the light emitted by the active waveguide film and traveling along it. This aspect is more important in devices with the resonator located in a separated layer than in those with modulated active film thickness. This is because in the former type, the electric field intensity is lower inside the grating and it decreases rapidly from the top surface of the active layer. According to that, it is expected that the existence of a residual layer would be detrimental for the device performance^31^. Figure 5a shows the electric field intensity distribution of the TE_0_ mode corresponding to *s* = 0 in the series of lasers with *s*_0_ = 400 nm where these effects are more clear. Note that the DFB resonator provides the feedback for the in-plane light propagation, as well as the way to extract the light out of the device, and both mechanisms depend on the interaction between the evanescent wave of the light traveling along the active film and the grating. Thus, in order to get insights into this we have defined an overlap factor *r* for measuring the strength of this interaction. We define the overlap factor, *r*, as the ratio between the area *A*_1_ under the curve in the grating corrugation region and the area *A*_0_ of a rectangle of width equal to *d* and height equal to electric field intensity at the active layer surface (see Fig. 5b). Then

2 $$r=\frac{{A}_{1}}{{A}_{0}}=\frac{{d}_{{\rm{eff}}}}{d}$$

where *d*_eff_ is the effective grating depth. According to this definition, *d*_eff_ is the width of the rectangle with a height equal to electric field intensity at the active layer surface, and area *A*_1_. The *r* and *d*_eff_ parameters obtained for the devices with no residual layer for the four series of lasers prepared (devices located with a dashed line in Fig. 4) are listed in Table 1 and its dependence on the grating depth shown in Fig. 5c. Note that equation (2), applicable to devices with *s* = 0, can be easily generalized to treat lasers with *s* > 0 by simply replacing in such equation the parameter *d*, by the amount (*d* + *s*).

**Table 1:** Parameters of DFB lasers with top-layer resonator architecture without residual layer (*s* = 0) and different grating depths (*d*).

*h*_DCG-0_^a^ (nm)	*d*^b^ (nm)	*E*_th_^c^ (μJ/cm^2^)	*LSE*^d^ (%)	*r*^e^	*d*_eff_^f^ (nm)
50	46	23	0.8	0.65	30
100	86	24	0.95	0.49	42
240	216	46	1.1	0.29	63
400	367	63	1.2	0.19	69

^a^Initial DCG layer thickness (error ± 5 nm);

^b^Grating depth (error ± 5 nm);

^c^DFB laser threshold (error ≈ 10%);

^d^Output laser efficiency (error ≈ 10%);

^e^Overlap factor, defined by considering the electric field intensity distribution corresponding to the grating as illustrated in Fig. 5b;

^f^Effective grating depth, defined as the product of the overlap factor and the grating depth.

The relationship between *d* (and *d*_eff_) with the laser threshold and with the *LSE*, for the devices (among the ones prepared) with no residual layer, is analysed through Fig. 5d,e, respectively. The laser threshold dependence on *d* in terms of the overlap factor *r* is rather complicated. This is because an increase of *d* increases external losses but also affects the feedback mechanism and, consequently, the net gain and the threshold. As observed in Fig. 5d, the threshold is maintained approximately constant in the lowest value (*E*_th_ ~ 25 μJ/cm^2^) for *r* > 0.4, which corresponds to grating depth values *d* < 130 nm (see Table 1 and Fig. 5c). Then, for *r* < 0.4 (*d* > 130 nm) the threshold increases rapidly. Literature about the influence of grating depth on the performance of DFB lasers is scarce and not conclusive because different device architectures are used. Huang *et al*.^42^ compared two DFB lasers with top-layer resonators with grating depths of 130 and 60 nm, and found, at variance with the results obtained in the present study, the lowest threshold in the one with larger depth (80 μJ/cm^2^, versus 150 μJ/cm^2^). There are more detailed studies with lasers based on resonators integrated in the active film. For example, in devices with gratings imprinted on fused silica and active films deposited on top, the lowest thresholds and larger laser efficiencies were obtained with the shallower grating depths^31,43^. More recently Doring *et al*.^32^ observed, in devices with relatively small depths (20–80 nm), that the laser threshold decreased only slightly with increasing corrugation height. For such devices the overlap factors, as defined in our work, would be *r* > 0.4 for which we have found that threshold is similar. Nevertheless, comparisons with these lasers should be taken with caution because for them, the active film thickness is modulated, so the variation of the grating depth implies a change in film thickness, which also has an influence on the threshold.

The analysis of the grating depth dependence of the *LSE* is simpler (see Fig. 5e). As observed, the *LSE* increases linearly with *d*_eff_. Such dependency supports the suitability of the overlap factor definition, given that *LSE* is known to be proportional to the available *η* (represented by *d*_eff_). Note that the *LSE* also increases with *d*, although non-linearly, first at high rate and then at slow rate presumably reaching a maximum value (see the curve linking data points for devices with *s* = 0 in Fig. 4b). A further increase of *d* appears to be useless, indicating that improving feedback seems to compensate the increasing out-coupling losses for small grating depths meanwhile out-coupling losses is the dominant mechanism for large grating depths. According to the previously discussed grating depth dependence of the laser threshold, for *d* ~ 130 nm (*r* ~ 0.4), which corresponds to approximate limiting *d* value to keep *E*_th_ < 25 μJ/cm^2^, the *LSE* is around 80% of its maximum possible value (see Fig. 5c). By increasing *d* up to around 150 nm, the *LSE* could be improved (up to 88% of its maximum value), but this would be in detriment of the laser threshold, which would increase up to 35 μJ/cm^2^.

### Effect of varying *s* and *d* on the laser wavelength

**Figure 6:**
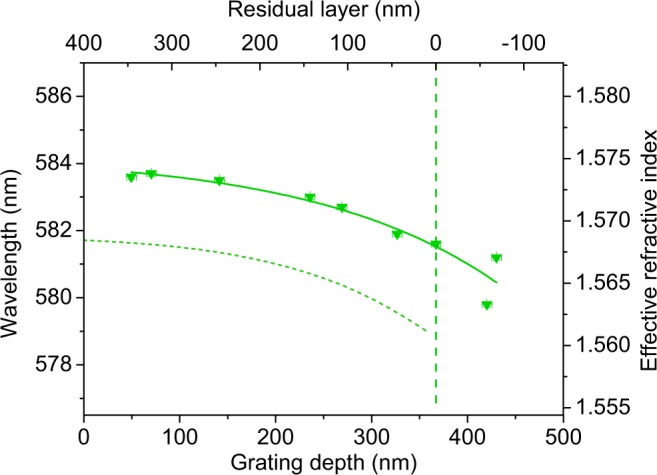
Influence of the grating depth on the laser wavelength. Experimental emission wavelength *λ*_DFB_ (triangles) as a function of the grating depth *d* (bottom axis) and the corresponding residual layer thickness *s* (top axis) for lasers with initial DCG layer thickness *s*_0_ = 400 nm. The full line is a guide to the eye. The dotted line corresponds to the resonant Bragg wavelength *λ*_Bragg_ calculated from Eq. 1 using experimental grating period Λ and effective index *n*_eff_ obtained from simulation (values in the right axis). The dashed vertical line indicates the resonator with no residual layer (*s* = 0), for which *d* = 367 nm.

The dependence of the experimental laser wavelength *λ*_DFB_ on the grating depth *d* is analysed through the results shown in Fig. 6 for the series of devices with *s*_0_ = 400 nm. A similar behaviour is observed for the other series (data for lasers with *s*_0_ = 240 nm shown in Supplementary Fig. 1, Supporting Information). The observed decrease in *λ*_DFB_ with increasing *d* is because the effective index *n*_eff_ of the structure (values in the vertical right axis of Fig. 6) decreases, as a consequence of the DCG material loss during development.

In order to get insights on the physical mechanism contributing to lasing, we compare the experimental *λ*_DFB_ values of the devices with residual layer *s* ≥ 0, to the corresponding resonant wavelengths *λ*_Bragg_ (calculated through Eq. 1 with the *n*_eff_ values obtained by simulation and plotted as a dashed line in Fig. 6). It is seen that in all cases emission occurs at a wavelength *λ*_DFB_, a few nm larger than *λ*_Bragg_. The observation of lasing at *λ*_DFB_ values above *λ*_Bragg_ is an expected result for top-layer resonator devices with *s* ≥ 0, because index coupling is the only mechanism responsible for the laser process^34,35^. Note that for DFB lasers with modulated active film thickness (i.e. devices with the active film deposited over a grating or with the grating directly imprinted on it, such as top-layer resonator DFB devices with *s* < 0), in addition to index coupling, there would be also a gain coupling contribution to lasing^31,44,45^. It is known that for a system with pure (or dominating) gain coupling, emission would occur at *λ*_Bragg_. The fact that in lasers with top-layer resonators with *s* ≥ 0 there is only one mechanism, makes them ideal systems to study the influence of the index coupling strength on the separation between *λ*_Bragg_ and *λ*_DFB_. For the devices analysed in Fig. 6 the separation between *λ*_DFB_ and *λ*_Bragg_ varies from around 2.1 nm (for the lasers with smaller *d*) up to 2.7 nm, for the device with no residual layer (*s* = 0) whose *d* = 367 nm. The observed increase in the separation between *λ*_DFB_ and *λ*_Bragg_ as *d* increases is because the strength of the index coupling process (which is proportional to the ratio *d*/*h*) gets larger. This phenomenology was clearly observed in devices with gratings imprinted on the substrate, in which the ratio *d*/*h* varied in a wide range among the different devices (between 0.2 and 1.9) because film thickness was also varied^31^. In the present case, for a given series of devices (all with the same *h*), the separation between *λ*_DFB_ and *λ*_Bragg_ changes only slightly as *d* increases, because the variation of the amount *d*/*h* is small. This effect is also seen with the data of the series of devices with *s*_0_ = 240 nm (Supplementary Fig. 1, Supporting Information). In that case, for the laser with no residual layer (*s* = 0) and *d* = 216 nm, the amount (*λ*_DFB_ − *λ*_Bragg_) = 1.4 nm. This value is smaller than the one previously discussed for the device with *s* = 0 and *d* = 367 nm, corresponding to the series of *s*_0_ = 400 nm, for which the amount (*λ*_DFB_ − *λ*_Bragg_) = 2.7 nm. This is in accordance with the strength of the index coupling mechanism (driven by *d*/*h*) for each series.

## Conclusions and Perspectives

The emission properties of organic DFB lasers with both, active film and resonator based on solution-processed polymeric materials and with the resonator located on top of the active film, have been studied as a function of two resonator parameters: the thickness of the residual photoresist layer and the grating depth. It was found that a fine control of these parameters is crucial for the design and fabrication of devices with optimized performance. The lowest laser thresholds and largest efficiencies correspond to resonators without residual layer (*s* = 0). As for the grating depth, both the threshold and the laser efficiency increase with the grating modulation depth, and for the latter, above a certain *d* value, a further increase is inefficient. Results for lasers with *s* = 0 and different gratings depth have been successfully interpreted in terms of the overlap between the electric field intensity distribution and the grating, through the definition of an overlap factor *r*. The lasing threshold decreases rapidly when *r* increases and is maintained approximately constant in the lowest value (*E*_th_ ∼ 25 μJ/cm^2^) for *r* > 0.4, corresponding to grating depths *d* < 120 nm. On the other hand, the *LSE* increases with decreasing overlap factors, but at large grating depths, the growth rate diminishes, and the *LSE* reaches a maximum value. According to this trade-off for optimizing simultaneously the threshold and the efficiency through the grating depth, a reasonable criterium is to choose depths in the range 100–130 nm (*r* ~ 0.5−0.4). Such lasers would have thresholds *E*_th_ < 25 μJ/cm^2^ and *LSE* ∼ 80% of its maximum possible value.

From the point of view of the DCG resonator processing, the dry development step to obtain the relief gratings has been simplified considerably, with respect to methods previously used. Here it has been done with an inexpensive plasma cleaner machine, in contrast to a much more sophisticated methods previously used (an oxygen electron cyclotron resonance stream inside a high vacuum chamber). Despite the isotropy of the process used in this work, the gratings have sufficiently good quality. The method is simple and inexpensive and should be useful in mass production of organic DFB lasers.

Finally, it should be remarked that the knowledge achieved through this work might have an important impact for plastic optoelectronic in a broader sense than the one explored here related to organic lasers. Note that these relief gratings might be used for other active laser materials, not necessarily organic, with the only requirement to be insoluble in water; or for other optoelectronic devices, such as light emitting diodes^46^ or solar cells^47^, with the purpose of improving their efficiency to extract or collect light. An example of a class of materials for which the resonators reported here might have applications are perovskites. Despite the great progress in the use of these materials for photovoltaic and LED applications^21–23,28^, the advances for lasing purposes, and more particularly in the form of DFB devices, has been more limited^25,26^. One of the reasons is the difficulty to prepare the perovskites as very thin (typically < 150 nm) optical waveguides with low scattering losses and low roughness and simultaneously keeping a high photoluminescence efficiency. Thus, it is key to optimize the synthesis for tight control of the grain size of the polycrystalline perovskite on the nanoscale, which requires synthetic methods rather different than the ones used with other kind of devices (e.g. solar cells)^25,26^. With regards to the potential use with perovskites of the DCG gratings reported here, a limitation of the top-layer configuration is the fact that the DCG layer is processed from water. This would require protection of the perovskite film, before depositing the DCG layer on top of it, for example by means of a protective polymer layer. On the other hand, the use of the DCG gratings in the configuration with the resist below the active film should be feasible, given that the DCG layer after development is water resistant. Ongoing work in these directions are presently being carried out in our laboratories.

Another research field in which the reported gratings might be of interest is that of two-dimensional (2D) materials. In recent years, the study of such systems in photonic devices of various kinds has aroused widespread attention due to their strong light–matter interaction^27,48–52^. A variety of 2D materials, such as graphene-like systems^48^, Tellurium nanosheets^49^ or perovskites nanosheets^27^, have demonstrated a good potential for non-linear optics devices. Particularly interesting for the purpose of the present work are recent reports of enhanced spontaneous emission and lasing on 2D Metal-chalcogenides^50–52^. Also promising is the use of nanographene nanosheets, whose use as DFB lasers with top-layer resonators has been just demonstrated^16^.

## Methods

### Device design and fabrication

Fabrication of 1D DFB lasers with the resonator on the top of the active layer consisted of the following steps: (1) Active film deposition: polystyrene (PS) films with the laser dye perylene orange (PDI-O) dispersed at 1.0 wt%, were deposited by spin-coating over commercial 2.5 × 2.5 cm^2^ transparent fused silica substrates (i.e. quartz plates) from a toluene solution. (2) Photoresist deposition: layers of the negative photoresist DCG were spin-coated over the active films from a hot water solution (40 °C). The concentration of gelatine (Russelot, 200 bloom) in the solution was varied between 0.8 wt% and 7 wt% in order to obtain layers of thickness in the range 50–400 nm. The sensitizer (ammonium dichromate) was dissolved in a proportion 35 wt% of gelatine. (3) Grating recording: one dimensional gratings were recorded by HL with light from an Ar laser emitting at 364 nm in a simple and stable setup in which a mirror is attached with a 90° angle to the sample holder^53^. Intensities of the interfering beams were equal in order to achieve the maximum contrast. The average exposure was 45 mJ/cm^2^ and an absorbent plate was attached to the back side of the substrate with an index matching liquid to avoid backward reflections. (4) Development: after desensitizing the DCG layer in a cool water bath (10 °C), surface-relief gratings were obtained by dry development in an oxygen plasma using the surface treatment machine Diener Zepto.

For the active film preparation, the amount of solvent was adjusted to obtain thickness of *h* ~ 600 nm, so the obtained waveguide supports one transversal electric mode (TE_0_) which propagates with a high confinement factor, thus minimizing losses and optimizing the amplified spontaneous emission (ASE) performance^54^. The concentration of PDI-O was chosen to be 1 wt% because it was previously shown that the ASE performance is optimized with this proportion^55^, thus enabling to prepare DFB lasers with low thresholds and long lifetimes^7,13^.

The grating period value was chosen to have laser emission (*λ*_DFB_) as closest as possible to the wavelength of maximum gain (i.e. that at which ASE occurs, *λ*_ASE_ = 579 nm). This is known to be crucial to optimize the threshold^31,38^. The calculation of the proper Λ to obtain emission at a given wavelength (once film thickness *h* was decided to be 600 nm), was done by calculating *n*_eff_ for the TE_0_ waveguide mode traveling in the active film, and the corresponding *λ*_Bragg_ value via eq. (1). Calculations of the *n*_eff_ values, were performed by means of a free-access software program (1-D mode solver for dielectric multilayer slab waveguides)^56^. The DCG resonator was simulated by two layers: one of uniform thickness *s* and the refractive index of the DCG; and the other one, of thickness *d*, and a refractive index equal to the weighted average index of the two media at each side of the corrugated surface (air and DCG). The refractive index values used in the calculations (at *λ* = 580 nm) were: 1.55, for exposed and developed DCG; 1.592 for dye-doped PS, which corresponds to the value of non-doped PS; and 1.459 for fused silica. Since Λ values for the various devices are between 371 and 372 nm and the error in grating fabrication is of the order of 2 nm, we have considered that Λ = 372 nm in all cases.

### Morphological characterization of the gratings

Grating periods were measured by comparing the corresponding diffraction pattern to those of calibrated reference gratings. The grating depth (*d*) and thickness of the residual layer (*s*) could be estimated from the initial thickness of the DCG layer (*s*_0_), i.e. the thickness before grating fabrication; and from the selectivity of the process. The selectivity, defined as the ratio of the development rate of non-exposed to the exposed areas, at the illumination dose used (45 mJ/cm^2^), was approximately 10. Grating profile studies were performed by means of FESEM (Carl Zeiss, model Merlin VP compact). Accurate determination of *d* variations was made by measuring the grating diffraction efficiency (*η*) and comparing it to results predicted by the coupled-wave theory taking into account the grating profile^57^. See data and details in Supplementary Fig. 2, Supporting Information.

### Optical characterization: refractive index and thickness

The refractive index and thickness of the active films and of the initial DCG layers have been determined by a modification of the envelope (or Swanepoel) method^58^. These methods are based on the analysis of interference fringes observed in the film transmission spectrum due to the light reflections in the film interfaces. The modified method used here allows to measure very thin films (thickness between 40 and 400 nm) in contrast to the standard envelope method, which is limited to values above 400 nm.

### Laser characterization

Laser characterization was performed under excitation with a frequency-doubled Neodimium Yttrium Aluminium Garnet, Nd:YAG laser (10 ns, 10 Hz,) emitting at 532 nm, as in previous studies^59^. The pump beam was linearly polarized parallel to the grating lines and incident at a 20° angle. The beam section over the sample was elliptical, with a minor axis of 1.1 mm. The emitted light was collected in reflectance, perpendicularly to the surface with an Ocean Optics USB2000 fiber spectrometer (resolution 1.3 nm) placed at about 1 cm from the sample. For spectral shape inspection a MAYA spectrometer (0.13 nm resolution) was used. The energy of the pulses was varied using neutral density filters.

The laser threshold (*E*_th_) was estimated as the minimum intensity at which laser emission occurs. The accuracy of the determination was increased by considering the abrupt change of linewidth defined as the full width at half of the maximum (FWHM), which accompanies the phenomenon. The absolute laser efficiency (*LSE*), defined as the ratio between laser output energy and incident pump energy and usually expressed as a percentage, was determined from the slope of a linear fit of the representation of the output energy versus the pump energy. The setup was similar than the one described above, but here the pump and emitted energy were measured with high resolution energy detectors Ophir PD10-C and PD10-pJ-C (resolutions of 1 and 0.01 nJ, respectively).

## Supplementary information

Supplementary information file

## Data Availability

The source data underlying Figs 2b, 3a–c, 4a,b, 5a,c-e and 6, as well as Supplementary Figs 1 and 2, are provided as Source Data files in the Institutional Repository of the University of Alicante [http://hdl.handle.net/10045/93108]. Other data supporting the findings of this manuscript are available from the corresponding authors upon reasonable request.
