# Supplementary material for: Controlling the emission properties of solution-processed organic distributed feedback lasers through resonator design
Source: Sci Rep. 2019 Aug 1;9:11159. doi: 10.1038/s41598-019-47589-4 (PMC6671999; doi:10.1038/s41598-019-47589-4)
Supplement: Supplementary file 1 — Supplementary information file [file 41598_2019_47589_MOESM1_ESM.pdf]

Bonal *et al.*

Supplementary Information

Controlling the emission properties of solution-processed organic distributed feedback lasers through resonator design

Víctor Bonal,[a] José A. Quintana,[b] José M. Villalvilla,[a] Pedro G. Boj,[b] and María A. Díaz-García [a]

[a] *Dpto. Física Aplicada, Instituto Universitario de Materiales de Alicante y Unidad Asociada UA-CSIC, Universidad de Alicante, 03080 Alicante, Spain.*

[b] *Dpto. Óptica, Instituto Universitario de Materiales de Alicante y Unidad Asociada UA-CSIC, Universidad de Alicante, 03080 Alicante, Spain.*

Correspondence and requests for materials should be addressed to: Prof. María A. Díaz-García (e-mail: maria.diaz@ua.es)

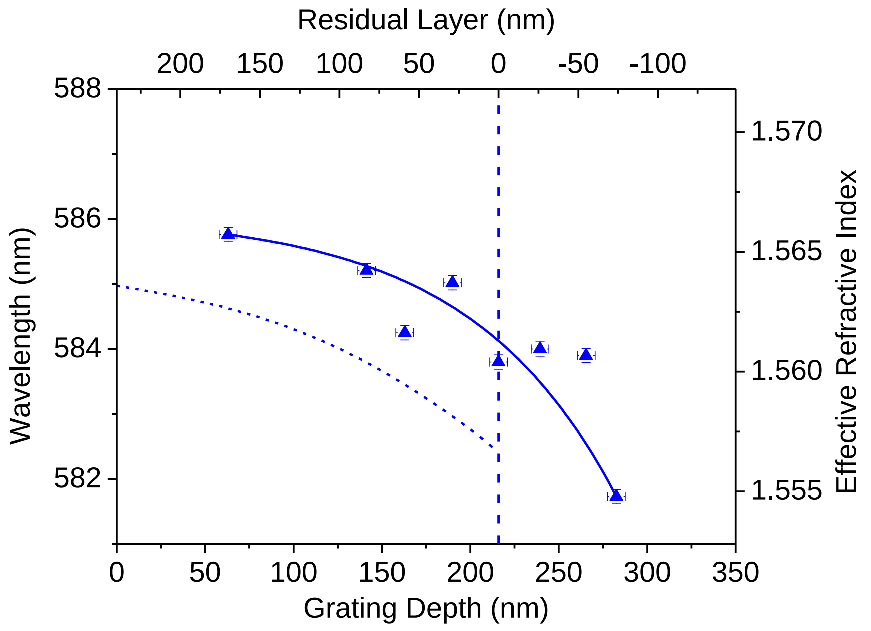

Supplementary Figure 1. **Influence of the grating depth on the laser wavelength.** Experimental emission wavelength λ_{DFB} (triangles) as a function of the grating depth d (bottom axis) and the corresponding residual layer thickness s (top axis) for lasers with initial DCG layer thickness s_0 = 240 nm. The full line is a guide to the eye. The dotted line corresponds to the resonant Bragg wavelength λ_{Bragg} calculated from Ec. 1 using experimental grating period Λ and effective index n_{eff} obtained from simulation (values in the right axis). The dashed vertical line indicates the resonator with no residual layer (s = 0).

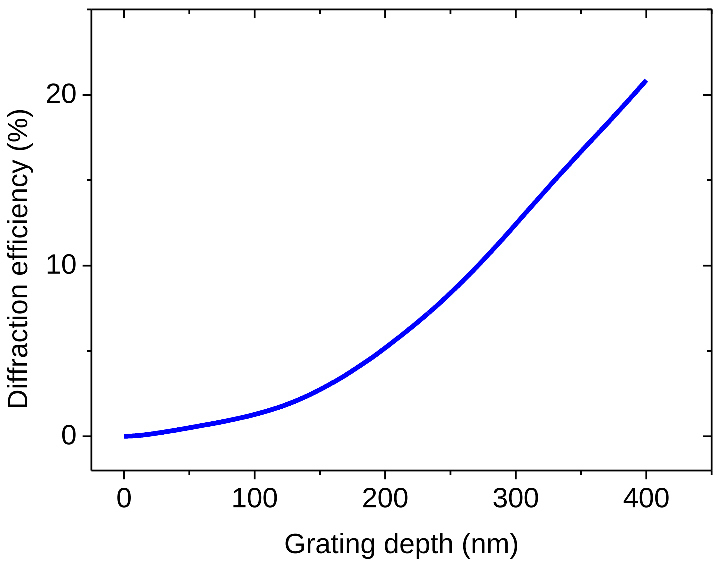

Supplementary Figure 2. Simulation of diffraction efficiency as a function of grating depth. Simulation of diffraction efficiency vs grating depth for a DCG layer grating with a period of 373 nm and duty cycle of 75:25 (hill:valley) illuminated at 29º with a wavelength of 364 nm. This curve obtained with the program Gsolver was used to translate experimental diffraction efficiencies in grating depth values.
